# Increased immunosuppression impairs tissue homeostasis with aging and age-related diseases

**DOI:** 10.1007/s00109-020-01988-7

**Published:** 2020-10-06

**Authors:** Antero Salminen

**Affiliations:** Department of Neurology, Institute of Clinical Medicine, University of Eastern Finland, P.O. Box 1627, FI-70211 Kuopio, Finland

**Keywords:** Aging, Alzheimer’s, Immunometabolism, Immunosenescence, Nitric oxide, Tissue degeneration

## Abstract

**Abstract:**

Chronic low-grade inflammation is a common hallmark of the aging process and many age-related diseases. There is substantial evidence that persistent inflammation is associated with a compensatory anti-inflammatory response which prevents excessive tissue damage. Interestingly, the inflammatory state encountered with aging, called inflammaging, is associated with the anti-inflammaging process. The age-related activation of immunosuppressive network includes an increase in the numbers of myeloid-derived suppressor cells (MDSC), regulatory T cells (Treg), and macrophages (Mreg/M2c). Immunosuppressive cells secrete several anti-inflammatory cytokines, e.g., TGF-β and IL-10, as well as reactive oxygen and nitrogen species (ROS/RNS). Moreover, immunosuppressive cells suppress the function of effector immune cells by catabolizing l-arginine and tryptophan through the activation of arginase 1 (ARG1) and indoleamine 2,3-dioxygenase (IDO), respectively. Unfortunately, the immunosuppressive armament also induces harmful bystander effects in neighboring cells by impairing host tissue homeostasis. For instance, TGF-β signaling can trigger many age-related degenerative changes, e.g., cellular senescence, fibrosis, osteoporosis, muscle atrophy, and the degeneration of the extracellular matrix. In addition, changes in the levels of ROS, RNS, and the metabolites of the kynurenine pathway can impair tissue homeostasis. This review will examine in detail the harmful effects of the immunosuppressive cells on host tissues. It seems that this age-related immunosuppression prevents inflammatory damage but promotes the tissue degeneration associated with aging and age-related diseases.

**Key messages:**

• Low-grade inflammation is associated with the aging process and age-related diseases.

• Persistent inflammation activates compensatory immunosuppression with aging.

• The numbers of immunosuppressive cells increase with aging and age-related diseases.

• Immunosuppressive mechanisms evoke harmful bystander effects in host tissues.

• Immunosuppression promotes tissue degeneration with aging and age-related diseases.

## Introduction

The remodeling of the immune system including the presence of chronic low-grade inflammation is one hallmark of the aging process [1, 2]. This age-related immune state has commonly been called inflammaging. Systemic chronic inflammation has also been implicated in many age-related diseases, aggravating the pathology of these diseases, e.g., cardiovascular diseases, chronic kidney disease, non-alcoholic fatty liver diseases, and neurodegenerative diseases [3]. Currently, the molecular origin of age-related inflammation needs to be clarified. Nonetheless, there is substantial evidence that persistent inflammation is associated with a compensatory anti-inflammatory response which prevents excessive tissue damage in the conditions of chronic inflammation [4, 5]. Interestingly, inflammaging is also associated with the anti-inflammaging process [6, 7], i.e., not only is there an increased level of anti-inflammatory cytokines but there is also clear evidence that the numbers of several immunosuppressive cells are augmented during the aging process [8] (see below). Similar effects have been observed in many age-related diseases, e.g., in atherosclerosis [9] and non-alcoholic fatty liver disease [10]. Immunosuppressive cells possess diverse immunosuppressive mechanisms which suppress the function of effector immune cells in inflamed tissues. However, only a few of these activities are specifically targeted to immune cells but in contrast, they can induce harmful bystander effects in the neighboring cells of host tissues. For instance, TGF-β, an anti-inflammatory cytokine, secreted by many immunosuppressive cells, can provoke degenerative changes in tissues which are similar to those induced by the aging process (see below). Moreover, suppressor cells induce a shortage of some amino acids in inflamed tissues which affects not only the immune cells but also the cells of host tissues. I will thoroughly examine the possibility that the activation of immunosuppressive cells causes degenerative bystander effects in host tissues with aging.

## Chronic inflammation provokes compensatory immunosuppression

Severe inflammatory conditions, e.g., autoimmune diseases, pathogen-induced sepsis, and traumatic injuries, induce a systemic state called the systemic inflammatory response syndrome (SIRS) [5, 11]. The SIRS-related disorders are associated with a compensatory anti-inflammatory syndrome (CARS) which prevents detrimental multiple organ failure [5, 12]. The life-threatening SIRS/CARS condition has also been termed the persistent inflammation, immunosuppression, and catabolism syndrome (PICS). It seems that the immunosuppressive CARS state can concomitantly appear with the pro-inflammatory SIRS phase although in many disorders, e.g., in sepsis and trauma, the SIRS condition dominates during the early phase, whereas immunosuppressive responses control the later phases [5, 13]. The common characteristics of CARS involve (i) the induction of emergency myelopoiesis, especially myeloid-biased hematopoietic stem cell differentiation, (ii) the excessive expansion of myeloid-derived suppressor cells (MDSC) and regulatory T cells (Treg), and (iii) the increased expression of anti-inflammatory cytokines in both circulation and affected tissues [5, 13]. Given that inflammatory factors are the main inducers of immunosuppressive cells, e.g., MDSCs [14, 15], we can expect that an excessive inflammation of SIRS state stimulates the immunosuppressive CARS response to counteract the detrimental inflammatory injuries occurring in host tissues.

**Fig. 1:**
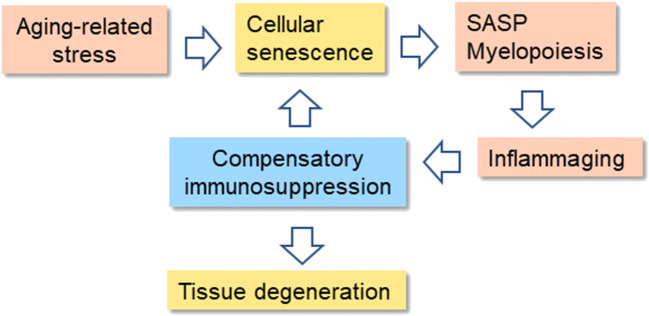
The feed-forward regulation between cellular senescence, inflammaging, and compensatory immunosuppression in the aging process. Several age-related stresses induce the accumulation of senescent cells into tissues. The pro-inflammatory phenotype (SASP) of senescent cells enhances the myelopoiesis and the recruitment of immune cells into aging tissues. The inflammaging state evokes compensatory immunosuppression which counteracts the low-grade inflammation present in aged tissues. Consequently, immunosuppression impairs the clearance of senescent cells, i.e., it enhances inflammaging and immunosuppression. Increased immunosuppression with aging impairs the maintenance of tissue homeostasis and induces the degenerative changes evident in aging tissues

There is abundant evidence that it is not only severe systemic inflammation which induces compensatory immunosuppression but also local immune activation can induce the stimulation of immunosuppression, e.g., in tumors and tissue transplantation [4, 16, 17]. In particular, compensatory immunosuppression is associated with non-resolving inflammatory states, e.g., autoimmune diseases, atherosclerosis, and tumors. In this respect, the aging process and Alzheimer’s disease can be considered as non-resolving conditions even though the persistent perpetrator is unknown. In tumors, chronic inflammation creates an immunosuppressive microenvironment, e.g., involving the activation of MDSCs and Tregs, which allows the escape of tumor cells from immune surveillance [16]. Interestingly, senescent cells, present in both tumors and aged tissues, secrete several inflammatory factors [18–20]. Senescent cells are most likely an important source of chronic inflammation in aged tissues since they are able to modify the phenotypes of immune cells and thus enhance immunosuppression in tumors and aged tissues (see below) (Fig. 1).

## Immunosuppressive network

The microenvironment has a critical role in the regulation of immune cell populations and their functions in tissues. Immune cells possess a great plasticity to modify their phenotypes and activities according to the changes in their microenvironment. Inflammatory responses linked to tissue injuries, infections, and tumors are potent inducers of the changes in immune homeostasis. For instance, in acute inflammation, macrophages polarize towards the pro-inflammatory M1 subtype, whereas in chronic inflammatory conditions, they can display anti-inflammatory M2 properties [21]. Inflammation is an alarming response which not only controls the phenotype of tissue-resident immune cells but can also stimulate myelopoiesis in the bone marrow and recruit immune cells into inflamed tissues. Subsequently, the infiltrated immune cells, e.g., monocytes, can become differentiated into other subtypes of myeloid cells in response to environmental signals. Interestingly, the cells of host tissues can educate immune cells by secreting a diverse set of soluble factors. For instance, tumor cells are able to educate immune cells into immunosuppressive phenotypes, e.g., tumor-associated macrophages (TAM) [22]. However, this property is not unique to cancer cells since many other cells, normal or transformed, can educate immune cells, e.g., fibroblasts [23] and keratinocytes [24]. In addition, changes in the extracellular matrix can regulate the properties and activation level of immune cells [25]. The immune system is a co-operative network which can control the activities and phenotypes of immune cells on the basis of the microenvironmental conditions.

**Fig. 2:**
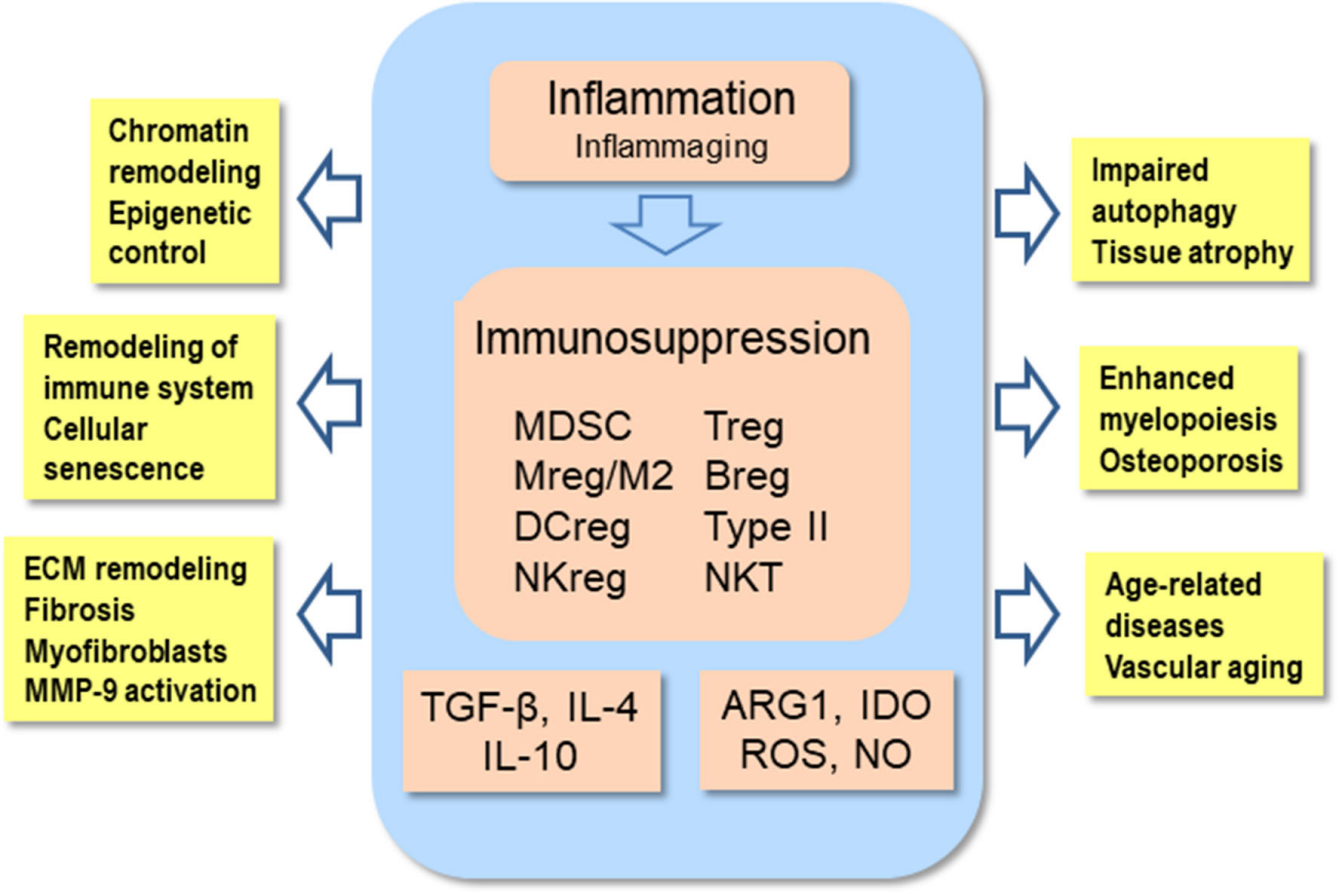
Chronic low-grade inflammation elicits compensatory immunosuppression by activating the cells of the immunosuppressive network. Immunosuppression is generated by the secretion of anti-inflammatory cytokines, e.g., TGF-β and IL-10, as well as through the release of ROS and NO. The activation of ARG1 and IDO enzymes induces the catabolism of l-arginine and tryptophan, respectively. Immunosuppression provokes many pathological changes in host tissues which are similar to those encountered during the aging process and in age-related diseases. ARG1, arginase 1; Breg, regulatory B cell; DCreg, regulatory dendritic cell; IDO, indoleamine 2,3-dioxygenase; IL, interleukin; MDSC, myeloid-derived suppressor cell; MMP-9, matrix metalloproteinase-9; Mreg/M2, regulatory macrophage; NKreg, regulatory natural killer cell; NO, nitric oxide; ROS, reactive oxygen species; TGF-β, transforming growth factor-β; Treg, regulatory T cell; Type II NKT, Type II natural killer T cell

Chronic inflammatory disorders remodel the phenotypes of immune cells towards the anti-inflammatory activities in inflamed tissues. The immunosuppressive phenotypes have been called regulatory subtypes since they can inhibit the functions of both myeloid and lymphoid effector cells [8, 17, 26]. This immunosuppressive network involves regulatory T (Treg) and B (Breg) cells of lymphoid lineage. Correspondingly, the immunosuppressive myeloid cells include myeloid-derived suppressor cells (MDSC) as well as regulatory macrophages (Mreg/M2c), dendritic cells (DCreg), natural killer cells (NKreg), and type II natural killer T cells (NKT) (Fig. 2). MDSCs are a heterogeneous group of immature myeloid cells which originate from myelopoiesis in the bone marrow, especially many inflammatory factors stimulate the generation of MDSCs [15, 17]. There are two types of MDSCs, i.e., the monocytic (M-MDSC) and polymorphonuclear (PMN-MDSC) subsets. Interestingly, MDSCs can be matured into other myeloid regulatory cells in inflamed tissues. Given that MDSCs secrete immunosuppressive factors, they can enhance or maintain the immunosuppressive properties of other regulatory cell types. Tregs are a heterogeneous group of T cells including thymic (tTreg), peripheral (pTreg), and inducible (iTreg) subtypes [27]. Tregs are the major immunosuppressive cells which can inhibit the functions of helper and cytotoxic T cells as well as those of B cells. They can also promote the generation of DCregs and M2 anti-inflammatory macrophages. Breg cells are also a diverse population of suppressive B cells which induce immunosuppressive processes by secreting IL-10 cytokines [28]. It is known that MDSCs promote the functions of Bregs [29], whereas Bregs promote the expansion of Tregs [30]. This illustrates well the co-operation between MDSCs, Tregs, and Bregs. The microenvironmental signals of inflamed tissues have a crucial role in the generation of other regulatory subtypes, i.e., DCregs, Mregs/M2c, and NKregs [8, 17, 26]. The immunosuppressive phenotypes of these regulatory myeloid cells have been augmented by the cytokines secreted by MDSCs, Tregs, and Bregs. Conversely, the suppressive subsets of myeloid cells inhibit their effector counterparts by secreting anti-inflammatory cytokines. Briefly, the members of immunosuppressive network co-operate to potentiate their suppressive activities in order to counteract the pro-inflammatory responses in chronic disorders (Fig. 2).

**Fig. 3:**
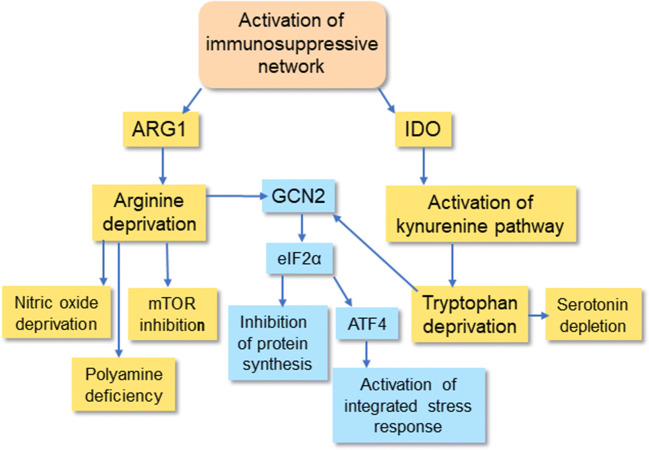
Amino acid catabolism caused by ARG1 and IDO triggers a shortage of l-arginine and tryptophan, disturbing the homeostasis of host tissues. IDO activates the kynurenine pathway which produces several toxic metabolites. GCN2/eIF2α/ATF4 signaling inhibits protein synthesis and activates the integrated stress response. ARG1, arginase 1; ATF4, activating transcription factor 4; eIF2α, eukaryotic translation initiation factor 2α; GCN2, general control non-depressible 2; IDO, indoleamine 2,3-dioxygenase; mTOR, mechanistic target of rapamycin

The immunosuppressive network possesses an armament of diverse mechanisms to enhance their own suppressive capacity which inhibits the functions of immune system in chronic inflammatory conditions. However, the targets are not only immune cells since the tools exert many bystander effects in the host tissues inducing the degeneration of tissues in chronic disorders. Anti-inflammatory cytokines secreted by regulatory immune cells are the key messengers which induce the immunosuppressive responses in inflamed tissues [31–33]. IL-10 and TGF-β are the major anti-inflammatory cytokines although there are some other immunosuppressive cytokines, e.g., IL-4, IL-11, IL-13, IL-35, and IL-1 receptor antagonist (IL-1ra). The secretion of the anti-inflammatory cytokines occurs in a context-dependent manner. Another important immunosuppressive mechanism is the increased expression of amino acid–catabolizing enzymes leading to a shortage of certain amino acids in inflamed tissues [34, 35] (Fig. 3). The robust induction of arginase 1 (ARG1) and indoleamine 2,3-dioxygenase (IDO) in suppressive cells consumes arginine and tryptophan, respectively, from the microenvironment which consequently inhibits protein synthesis and ultimately suppresses the proliferation of pro-inflammatory cells in chronic disorders. Moreover, any deficiency of arginine and tryptophan will also affect protein synthesis in the cells of host tissues. It is not only the shortage of amino acids but also the activations of ARG1 and IDO generate metabolites which disturb the metabolism of host tissues. Given that arginine is the shared substrate for ARG1 and nitric oxide synthase (NOS), the induction of ARG1 inhibits the generation of nitric oxide (NO) [36] (Fig. 3). Correspondingly, the activation of IDO stimulates the kynurenine pathway which is involved in the pathogenesis of many chronic diseases [37]. Although the activation of ARG1 and IDO has an indispensable role in the maintenance of immunosuppression, concurrently, it exposes host tissues to many detrimental bystander effects (Figs. 2 and 3).

## Cellular senescence promotes inflammaging and immunosuppression

There are a number of theories on the mechanisms driving the aging process, i.e., a spectrum of diverse mechanisms ranging from damage hypotheses to programmed theories. Recently, the roles of cellular senescence and the remodeling of the immune system have received considerable attention [2, 38]. An irreversible arrest of cell cycle is one major hallmark of cellular senescence although there are other common characteristics, e.g., resistance to apoptosis, flat cell morphology and nuclear changes, dysfunctions in mitochondria and the lysosomal system, and several alterations in the maintenance of proteostasis. Interestingly, senescent cells display a secretory phenotype and thus this state has been called the senescence-associated secretory phenotype (SASP) [39]. For instance, senescent cells not only secrete inflammatory mediators, e.g., interleukins, chemokines, and colony-stimulating factors, but also growth factors, proteases, and extracellular matrix proteins [19, 39, 40]. The secretion of exosomes is also increased from senescent cells [41, 42]. It seems that senescent cells secrete pro-inflammatory factors to alert the immune system about imminent danger and to enhance the elimination of senescent cells. It is likely that senescent cells displaying the SASP properties are the most important source of the inflammaging process which evokes the counteracting immunosuppressive response (Fig. 1).

### Senescent cells accumulate into tissues with aging

There is convincing evidence that senescent cells accumulate within tissues during the aging process [43, 44]. The in vivo biomarkers of senescent cells include an enhanced expression of p16INK4a and SA-β-Gal as well as the appearance of senescence-associated heterochromatin foci (SAHF). An increase in the numbers of senescent cells is also associated with many age-related diseases, e.g., age-dependent hepatic steatosis [45] and cardiovascular diseases [46]. Ogrodnik et al. [45] reported that senescent hepatocytes promoted the severity of age-dependent steatosis in mouse liver, whereas the suicide gene–mediated clearance of senescent hepatocytes reduced the accumulation of fat into mouse liver with aging. Interestingly, there are studies indicating that senescent cells can induce a form of bystander senescence of neighboring cells in a paracrine manner [47, 48]. The paracrine transmission of senescence may be mediated by exosomes [49] and soluble SASP components, e.g., IL-1α and TGF-β [50]. In addition to the enhanced production of senescent cells, it seems that the reduced clearance of senescent cells also augments the accumulation of senescent cells into aging tissues. Sagiv et al. [51] demonstrated that the surveillance and clearance of senescent human fibroblasts were mediated by the NKG2D receptors of NK cells. However, the accumulation of pro-inflammatory senescent cells into tissues evokes a counteracting response, i.e., the recruitment and expansion of immunosuppressive cells within these tissues (see above) [8]. It is known that MDSCs and Tregs can inhibit the cytotoxicity of NK cells [52, 53] thus preventing the clearance of senescent cells from aging tissues. It seems that there exists a feed-forward regulation between the accumulation of senescent cells and the age-related immunosuppression (Fig. 1).

### Inflammatory phenotype of senescent cells promotes inflammaging and immunosuppression

The inflammatory phenotype is a hallmark of senescent cells although the type of stress stimuli and the actual cells involved affect the profiles secreted by senescent cells [19, 38, 54]. For instance, Wiley et al. [54] reported that the mitochondrial dysfunction–associated senescence (MiDAS) did not induce the secretion of the cytokines of the IL-1 family in human fibroblasts and progeroid mice in vivo. The most secreted interleukins have included IL-6, IL-7, IL-1β, and IL-8 in the different models [19]. Senescent cells also robustly (over 4-fold) secrete colony-stimulating factors (CSF), e.g., GM-CSF and G-CSF. Interestingly, GM-CSF is a potent driver of chronic inflammation since it activates myelopoiesis and promotes the expansion and differentiation of MDSCs and Tregs [55, 56]. It is known that there is a significant increase in myelopoiesis with aging, whereas lymphopoiesis is downregulated [57]. Senescent cells also secrete chemokines and other chemoattracting molecules, e.g., CCL2, CCL3, CCL8, and CXCL1,2,3 (over 4-fold), which recruit myeloid cells into inflamed tissues [19]. A low-grade inflammation, i.e., inflammaging, is associated with a significant increase in the levels of many inflammatory mediators in peripheral tissues and circulation [2, 7, 40]. Benayoun et al. [40] demonstrated that the transcriptional trajectories of several innate immunity pathways were robustly increased with aging in many tissues of humans and rodents. In particular, the NF-κB and JAK/STAT signaling pathways were most evidently induced with aging. NF-κB signaling can be a driving force for both the proinflammatory and anti-inflammatory responses, e.g., promoting the differentiation and activity of MDSCs and Tregs [58, 59]. Moreover, JAK/STAT signaling is the major inducer of the differentiation and expansion of MDSCs [58]. It is not only the proinflammatory processes which increase with aging but also the anti-inflammatory responses, termed anti-inflammaging, are augmented during the aging process [6–8]. The situation is reminiscent of the compensatory immunosuppression encountered in inflammatory diseases (see above). It is also known that different cellular stresses, e.g., oxidative stress and ER stress, are potent inducers of inflammation which enhances the differentiation and expansion of immunosuppressive MDSCs [60–62]. Ruhland et al. [20] demonstrated that the upregulation of senescent stromal cells in mouse skin provoked inflammation which promoted the appearance of an immunosuppressive microenvironment, i.e., senescent cells augmented the levels of MDSCs and Tregs in mouse skin. They also reported that the senescent stroma in mouse skin decreased immune surveillance which enhanced tumorigenesis. Interestingly, the presence of senescent stromal cells was significantly increased in the skin of elderly people, concurrently with the accumulation of MDSCs. This indicates that the age-related increase of pro-inflammatory senescent cells might promote a compensatory immunosuppression which prohibits the clearance of senescent cells in both tumors and aged tissues.

### Evidence on the increased immunosuppression in the aging process

There are some difficulties encountered in determining whether compensatory immunosuppression is associated with the aging process and age-related diseases since immune cells are exceedingly plastic cells, i.e., they can become gradually polarized between the proinflammatory and anti-inflammatory phenotypes in a context-dependent manner [8, 63]. For instance, the anti-inflammatory cytokines, e.g., IL-10 and TGF-β, can induce the conversion of myeloid and lymphoid cells towards the immunosuppressive regulatory phenotypes, i.e., the network co-operates and potentiates its suppressive properties according to the demands of the microenvironment. In addition, there are many technical difficulties, e.g., in the application of flow cytometric techniques in tissue assays. However, there is convincing evidence that the numbers of MDSCs increase with aging in the circulation of humans and mice [64, 65]. Verschoor et al. [65] reported that the numbers of PMN-MDSCs were significantly increased in the circulation of both community-dwelling seniors (61–76 years, *n* = 45) and frail elderly people (67–99 years, *n* = 131) as compared with the PMN-MDSC level of healthy adults (19–59 years, *n* = 41). This phenomenon does not only occur in the circulation since it has been reported that the numbers of MDSCs were augmented with aging in mouse bone marrow, spleen, and lymph nodes [64, 66] as well as in human and mouse skin [20]. Flores et al. [66] demonstrated that the age-related increase in the presence of MDSCs in mouse bone marrow and spleen was induced by the activation of NF-κB signaling. They also revealed that the number of MDSCs was clearly increased in the bone marrow of the progeroid mouse mutants, *BubR1* and *Ercc1*. There is substantial evidence that the presence of Tregs clearly increases with aging in the blood and lymphoid tissues of humans and mice [67, 68] as well as in mouse visceral adipose tissue [69] and skin [20]. Lages et al. [67] reported that the frequency of FoxP3-positive Tregs was significantly higher in the circulation of elderly participants (over 70 years, *n* = 16) than in young adults (below 30 years, *n* = 16). In addition, the occurrence of immunosuppressive M2 macrophages was found to increase with aging in mouse bone marrow, spleen, lungs, and skeletal muscles [70, 71]. It is not only the number of suppressive cells but also their immunosuppressive activities that are enhanced with aging. For instance, Enioutina et al. [64] demonstrated that the MDSCs isolated from the lymphoid organs of old mice significantly inhibited the antigen-induced T cell proliferation in vitro and the T cell–dependent antibody responses in vivo. These observations indicate that the expansion of immunosuppressive cells with aging impairs the function of T cells. Recently, I have reviewed the literature concerning the age-related activation of immunosuppressive network [8].

## Immunosuppression impairs tissue homeostasis in chronic inflammation

The activation of immunosuppressive cells in chronic inflammation suppresses the functions of innate and adaptive immunity. In addition to the targeted mechanisms, e.g., checkpoint immune receptors, immunosuppressive cells secrete anti-inflammatory cytokines and ROS compounds to inhibit immune responses. Moreover, these cells can also prevent the functions of immune cells by catabolizing l-arginine and tryptophan and thus induce a local shortage of these amino acids in the affected microenvironments. Nonetheless, these non-targeted mechanisms not only suppress immune cells but also impair the homeostasis of host tissues and consequently, they can provoke degenerative changes similar to those observed in the aging process and age-related diseases (Fig. 2).

### Anti-inflammatory cytokines

#### TGF-β

The TGF-β family of mammals involves three TGF-β isoforms, i.e., TGF-β1, TGF-β2, and TGF-β3, which control both the SMAD-dependent and independent pathways via the activation of TGF-β receptors (TGF-βR1 and TGF-βR2) [31]. The expression of TGF-β cytokines as well as that of TGF-β receptors displays low tissue specificity (Human Protein Atlas) although the activated immunosuppressive cells are the major source of TGF-β cytokines in chronic inflammation. The activation of TGF-β signaling is also controlled by many accessory proteins, e.g., latency-associated peptides and distinct integrins [72]. TGF-β is a pivotal cytokine in the regulation of the variety of functions undertaken by both innate and adaptive immunity [31, 73]. Not only does TGF-β signaling regulate immune responses but it also controls cell fates during development and many pathological conditions, e.g., cellular senescence, fibrosis, and tumorigenesis. Because of the diversity of the signaling mechanisms, TGF-β can generate both beneficial and detrimental responses in a context-dependent manner [74]. Inflammatory mediators stimulate the secretion of TGF-β from many immune cells, e.g., Tregs, MDSCs, M2 macrophages, and dendritic cells produce TGF-β cytokines. TGF-β is a potent anti-inflammatory cytokine which enhances the resolution of inflammatory insults by suppressing immune activity, e.g., through the activation of immunosuppressive network [31, 73]. For instance, TGF-β affects the myeloid cells by (i) enhancing the switch of pro-inflammatory M1 macrophages towards their immunosuppressive M2 phenotype, (ii) decreasing the cytotoxicity of neutrophils and NK cells, and (iii) reducing antigen presentation by dendritic cells [73]. In particular, TGF-β signaling regulates the development of T lymphocytes in the thymus, promotes their differentiation into special T cell subtypes, and finally, controls their activities in diverse pathological conditions. For instance, TGF-β suppresses the functions of CD4 and CD8 T cells, whereas it stimulates the expression of the *FoxP3* gene in CD4 T cells and thus triggers their differentiation into immunosuppressive Treg cells [31]. TGF-β and IL-10 are the major immunosuppressive cytokines which not only suppress inflammatory responses but simultaneously potentiate the suppressive properties of other immune cells including the immunosuppressive network. This indicates that immunosuppressive cells act as a co-operative network which has an important role in chronic inflammatory conditions, e.g., in the aging process [8].

TGF-β signaling does not only suppress the functions of immune cells but it can also arrest the cell cycle and induce cellular senescence in different non-immune cells [75, 76]. For instance, TGF-β stimulated the expression of p16INK4a and p19ARF and subsequently induced the growth arrest and senescence of mouse keratinocytes [77]. There is substantial evidence that TGF-β stimulates the expression of cyclin-dependent kinase inhibitors (CDKI), e.g., p15, p16, p21, and p27, which consequently induce cellular senescence [75, 76]. TGF-β can also inhibit the expression of human telomerase (hTERT) and thus provoke the attrition of telomeres which subsequently triggers cellular senescence [75]. Rapisarda et al. [78] demonstrated that the integrin β3 subunit (ITGB3) activated TGF-β signaling and accelerated senescence in human primary fibroblasts. The expression of ITGB3 protein and the components of TGF-β signaling were abundantly expressed in fibroblasts derived from elderly humans. It is also known that TGF-β signaling stimulates the NF-κB-driven generation of the SASP state in many cell types [79]. The senescent cells exhibiting the SASP phenotype secrete diverse chemokine, cytokines, and colony-stimulating factors [19]. These inflammatory products are not only potent activators for the generation of myeloid suppressor cells, e.g., MDSCs, but they can also direct their recruitment into senescent tissues [80]. There can also appear age-related changes in the TGF-β signaling pathway. van der Kraan [81] revealed that there are age-related changes in the SMAD-dependent TGF-β signaling in articular cartilage, i.e., the protective SMAD2/3 signaling was shifted towards the degeneration-linked SMAD1/5/8 signaling which provoked the generation of osteoarthritis with aging. It is likely that senescent cells are the major source of chronic low-grade inflammation observed in aging tissues (see above). Cellular senescence is an important tissue remodeling mechanism, e.g., during embryonic development and tissue injuries [82], but it seems that with aging, the accumulation of senescent cells enhances tissue degeneration. Currently, senescent cells are a challenging target for the development of anti-aging drugs. For instance, Baker et al. [83] demonstrated that the clearance of p16INK4a-positive cells from the wild-type mice extended the lifespan of both male and female mice. They also revealed that the accumulation of p16INK4a cells enhanced the aging process in mouse cardiac muscle. It seems that TGF-β signaling has a significant role in the degeneration of tissues through the augmentation of cellular senescence in the age-associated chronic inflammation.

Fibrosis, i.e., an excessive accumulation of fibrous proteins in the extracellular matrix (ECM), is a typical pathological hallmark of aging in several organs, e.g., myocardium, kidney, lungs, and liver. There is convincing evidence that TGF-β signaling is the crucial regulator in the formation of fibrosis with aging [84] (Fig. 2). The signaling pathways through the activation of SMAD2 and SMAD3 factors induce the expression and subsequently the secretion of collagens, fibronectins, proteoglycans, and glycosaminoglycans into the ECM. Different cell types, e.g., myofibroblasts and epithelial cells, can evoke the TGF-β-induced secretion of fibrous proteins in a context-dependent manner. TGF-β signaling can also induce the differentiation of diverse cell types into myofibroblasts, e.g., through the process of epithelial/endothelial-mesenchymal transition (EMT) [85]. Chronic inflammation is a common cause of fibrosis; most probably this can be attributed to the activation of immunosuppressive network. For instance, anti-inflammatory M2 macrophages are involved in the formation of fibrosis in the kidney [86]. Lebrun et al. [87] revealed that MDSCs promoted lung fibrosis by secreting TGF-β1. Tregs also regulate lung fibrosis by controlling the activities of other T cells, e.g., Th17 cells [88]. Interestingly, there are studies indicating that the AMPK and Klotho signaling pathways, two potent anti-aging mechanisms, suppress the TGF-β-induced fibrogenic responses since they inhibit the signaling through the SMAD2/3 pathway [89, 90]. Doi et al. [89] demonstrated that the secreted ectodomain of Klotho protein bounds to the TGFβR2 protein, thus inhibiting the downstream signaling of TGF-β and consequently preventing renal fibrosis. Klotho protein has many therapeutic effects, e.g., in vascular pathophysiology [91], which might be elicited through the blockade of TGF-β signaling.

There is a close interaction between TGF-β signaling and the components of the ECM [92]. For instance, ECM proteins can control the activation of TGF-β signaling via integrin receptors [72]. Conversely, TGF-β signaling stimulates the expression of enzymes which remodel ECM structures, e.g., matrix metalloproteinase-9 (MMP-9) [93] and several collagenases (MMP-1/13) and stromelysins (MMP-3/10/11) [94]. Toba et al. [95] demonstrated that the overexpression of MMP-9 in mouse macrophages enhanced the age-related cardiomyocyte hypertrophy, induced chronic low-grade inflammation in the myocardium, and increased cardiac fibrosis. Secreted proteinases cleave the danger-associated molecular patterns from extracellular matrix (ECM-derived DAMPs), e.g., aggrecan, biglycan, and decorin, which consequently activate the receptors of innate immunity [96]. It seems that ECM-derived DAMPs can promote both pro-inflammatory and anti-inflammatory responses in a context-dependent manner. For instance, Shao et al. [97] demonstrated that the MMP-9-cleaved osteopontin (OPN) protein induced the expansion of mouse MDSCs which probably activated immunosuppressive network, as described above. Moreover, it is known that elastin-derived peptides enhance the pathogenesis of several age-related diseases, e.g., atherosclerosis [98]. Interestingly, ECM bioscaffolds are able to modulate immune responses, e.g., through macrophage polarization [99]. LoPresti and Brown [100] presented results indicating that the exposure of bone marrow macrophages to ECM biomaterials derived from the small intestine submucosa of aged pigs promoted the immune responses commonly induced by anti-inflammatory M2 macrophages. The interaction of TGF-β signaling and matrix metalloproteinases has a crucial role in the remodeling of ECM with aging which affects stem cell homeostasis, tissue regeneration, and cellular senescence [101]. There is clear evidence that the expression of MMPs increases with aging in many tissues, e.g., those of MMP-1, MMP-9, and MMP-10 in human dermis [102]. Correspondingly, there are observations indicating that the number of MDSCs is increased in the skin of elderly people [20]. It seems that the age-related remodeling of ECM can be induced by TGF cytokines secreted by immunosuppressive cells (Fig. 2). However, it is not known whether the disturbances in ECM might precede the accumulation of immunosuppressive cells into tissues.

The aging process increases myelopoiesis, whereas lymphopoiesis declines [57]. It is believed that this myeloid dominance in the activity of hematopoietic stem cells (HSC) is attributable to inflammaging since it is known that inflammatory mediators enhance myeloid-biased hematopoiesis [57, 103]. In inflammatory conditions, emergency myelopoiesis can also occur in sites other than in bone marrow, e.g., in the spleen and liver; this process has been called extramedullary myelopoiesis. TGF-β is abundantly present in hematopoietic sites but the role of TGF-β signaling in the age-related changes still needs to be clarified. Challen et al. [104] demonstrated that TGF-β1 was a stimulatory factor for myeloid-biased HSC proliferation and differentiation, whereas TGF-β1 inhibited the lymphoid-biased HSC clones in mice. This indicates that TGF-β regulates the lineage determination of HSC clones. Subsequently, Quere et al. [105] demonstrated that transcription intermediary factor 1γ (TRIF1γ), also known as tripartite motif-containing 33 (TRIM33), decreased the stability of TGFBR1 and thus suppressed TGF-β signaling. They revealed that the myeloid-biased HSCs superimposed the responses induced by TGF-β signaling since these clones displayed a reduced expression of TRIF1γ and correspondingly, there was an increased level of TGFBR1 protein. Quere et al. [105] reported that the expression of TRIF1γ declined in HSCs with aging which stimulated myelopoiesis. Interestingly, Flores et al. [66] revealed that there was an expansion of MDSCs with aging in mouse bone marrow. Given that MDSCs are immature myeloid cells, this emphasizes the role of TGF-β-induced myelopoiesis in the generation of immunosuppressive MDSCs in bone marrow. ECM disturbances in bone marrow with aging might also enhance the generation of MDSCs. Intriguingly, several studies have revealed that MDSCs can be differentiated into osteoclasts, both in vitro and in vivo [106, 107]. The osteoclasts derived from MDSCs are able to provoke bone resorption, e.g., in multiple myeloma [106] and collagen-induced arthritis [107]. Zoledronate, a potent inhibitor of bone resorption, was able to inhibit the expansion and differentiation of MDSCs into osteoclasts and subsequently it reduced bone lesions in myeloma-bearing mice [106]. It seems that the increased myelopoiesis associated with inflammatory changes in bone marrow induces the generation of MDSCs which enhance age-related osteoporosis (Fig. 2).

TGF-β signaling has a crucial role in several age-related diseases, e.g., muscle atrophy [108], skin aging [109], cardiovascular diseases [110], and Alzheimer’s disease [111]. Carlson et al. [112] observed that the expression of TGF-β as well as the activation of Smad3 (pSmad3) increased with aging in mouse skeletal muscle. Particularly, the activity of Smad3 was robustly increased in myofibers. Narola et al. [113] demonstrated that the muscle-specific overexpression of TGF-β1-induced myofiber atrophy and endomysial fibrosis in mouse skeletal muscles. In sarcopenia, TGF-β signaling provokes fibrosis and muscle atrophy by increasing the expression of atrogin-1 and MuRF-1, E3 ubiquitin ligases, thus enhancing protein degradation [108]. TGF-β signaling also promotes the aging process through the remodeling of the ECM in many tissues, e.g., in the aging skin [109]. Moreover, age-related changes in the ECM have a key role in vascular aging; coronary artery disease is a good example [110]. Especially, TGF-β affects the function of smooth muscle cells in arterial aging. TGF-β signaling controls the major risk factors for atherosclerosis, i.e., fibrosis, hyperlipidemia, hypertension, inflammation, vascular remodeling, and arterial calcification [114]. Interestingly, there are observations indicating that TGF-β enhances the cerebral amyloid angiopathy in Alzheimer’s disease (AD) [111, 115]. The expression of TGF-β was robustly increased in the brains of AD patients, especially in those microvessels that were accumulating β-amyloid proteins. In contrast, there are studies indicating that TGF-β signaling might be impaired in AD. For instance, Baig et al. [116] demonstrated that hyperphosphorylated tau protein disturbed the signaling of Smad2/3 in neurons thus promoting AD pathogenesis. This controversy might reflect the context-dependency of TGF-β responses in pathological conditions.

#### IL-10

The IL-10 family consists of nine members which have an important role in the regulation of tissue integrity and homeostasis by suppressing inflammation and enhancing tissue repair after injuries and infections [32]. In brief, IL-10 cytokines stimulate the signaling of STAT3 transcription factor through the receptor complex of IL-10R1 and IL-10R2 proteins. IL-10 signaling inhibits the production of inflammatory cytokines, colony-stimulating factors, and several chemokines in monocytes and macrophages. Sun et al. [117] reported that IL-10 signaling inhibited the activation of NLRP3 inflammasomes in a STAT3-dependent manner in mouse microglia. IL-10 is not only an anti-inflammatory cytokine but it also has major functions in adaptive immunity. For instance, IL-10 cytokine suppresses immune responses by inhibiting some functions of dendritic cells as well as those of T and B cells. Several studies have revealed that IL-10 signaling prevents antigen presentation by dendritic cells [118]. However, the effects of IL-10 cytokines are greatly context-dependent, e.g., within T cell subsets [119]. Interestingly, IL-10 cytokines are able to increase the differentiation and activity of immunosuppressive cells. For instance, Hsu et al. [120] demonstrated that IL-10 exposure increased the expression of FoxP3 protein in human CD4^+^ T cells and consequently augmented their differentiation into induced Tregs (iTreg). IL-10 treatment also increased the immunosuppressive activity of iTregs. Moreover, IL-10 cytokines have a significant role in the polarization of pro-inflammatory M1 macrophages towards their immunosuppressive M2 phenotypes, e.g., after mouse myocardial infarction [121]. Accordingly, immune suppressive cells, e.g., regulatory B cells (Bregs), possess a robust capacity to secrete IL-10 cytokines and thus inhibit the functions of monocytes, dendritic cells, and T cells [122]. Overall, IL-10 cytokine is a significant suppressor of inflammatory responses and an important enhancer of immunosuppressive network.

IL-10 cytokines also affect other cells than simply the immune cells since IL-10RA and IL-10RB receptor proteins are expressed in a wide variety of tissues (Human Protein Atlas). IL-10 activates the STAT3 signaling which controls many regulatory functions in cytoplasm, mitochondria, and nuclei. For instance, STAT3 signaling has an important role in autophagy, both in immune and non-immune cells [123, 124]. There is substantial evidence that IL-10 signaling is an important inhibitor of autophagy although in some contexts, it can promote autophagy [124, 125]. For instance, IL-10 signaling inhibited the autophagy in human fibroblasts derived from hypertrophic scars [126]. Shi et al. [126] demonstrated that the crosstalk between IL-10R-STAT3 and AKT/mTOR signaling pathways promoted the inhibition of autophagy in human fibroblasts. mTOR is a well-known inhibitor of autophagy. Moreover, Kishore et al. [127] reported that IL-10 inhibited the pathological autophagy in rat ventricular cardiomyocytes induced by angiotensin II treatment. They demonstrated that IL-10 controlled autophagy via the AKT/mTOR pathway in rat cardiomyocytes. The observation that IL-10 can prevent autophagy via the PI3K/AKT/mTOR pathway is interesting since it is known that insulin/IGF signaling inhibits autophagy through an activation of the mTOR complex which subsequently promotes the aging process [128]. However, it seems that the IL-10-induced inhibition is dependent on cell type and signaling connections since Ip et al. [125] reported that the lipopolysaccharide-induced IL-10 signaling suppressed mTOR activity and subsequently activated autophagy in human macrophages. Currently, IL-10 signaling is associated with only a few pathological processes as compared with that of TGF-β signaling. Halvorsen et al. [129] demonstrated that IL-10 promoted the formation of the oxidized LDL-induced foam cells in human acute coronary syndrome (ACS). IL-10 signaling enhanced lipid accumulation as well as increasing the expression of anti-apoptotic genes in the macrophages isolated from ACS patients. Nakamura et al. [130] observed that the aging process robustly increased the expression of IL-10 in mouse retina and spleen. They revealed that increased STAT3 activation induced the generation of immunosuppressive M2 macrophages which promoted angiogenesis in the eyes of old mice. The targeted inhibition of IL-10R’s function and STAT3 signaling prevented the pathological neovascularization. Interestingly, Chakrabarty et al. [131] demonstrated that the overexpression of IL-10 in transgenic APP mice significantly augmented the deposition of β-amyloid in the hippocampus and cortex. Accordingly, the overexpression of IL-10 evoked memory impairments in transgenic APP mice. Given that IL-10R proteins are widely expressed in tissues, it is likely that the IL-10 cytokine not only affects immune cells but it also triggers bystander effects in inflamed tissues.

Immunosuppressive IL-10 cytokines mediate their transcriptional responses via the activation of STAT3 signaling, whereas TGF-β cytokines induce their effects via the SMAD pathways (see above). It is known that both the STAT3 and SMAD factors modify the chromatin landscape in the promoter regions of their target genes [132, 133]. Epigenetic modification either enhances the transcription of the target gene or represses its activity. The regulatory loci of *IL-10* and *TGF-β* genes are recognized as being under the epigenetic regulation [134, 135]. In addition, it is known that STAT3/SMAD factors control the expression of chromatin modifiers and in that way, they can remodel the whole genome. For instance, IL-10 signaling remodels the chromatin landscape of adipose tissue to repress adipocyte thermogenesis [136]. Intriguingly, the *IL-10* locus can be specifically remodeled in immune cells and differentially modified in response to diverse signals [137]. TGF-β signaling regulates cellular senescence and cardiac aging through the epigenetic regulation of chromatin landscapes [138] (Fig. 2). The remodeling of gene expression patterns by modifying chromatin loci is especially important in chronic conditions, e.g., in the aging process and age-related diseases. Recently, Benayoun et al. [40] demonstrated that the aging process induced clear alterations in the epigenomic and transcriptomic landscape of mouse and human tissues, e.g., in the heart, liver, and cerebellum. Interestingly, they observed that aging robustly upregulated the markers of many immune response pathways. Thus, it seems that epigenetic regulation is the way to control the plasticity of the immune cells during the aging process.

#### Other cytokines

There are several other anti-inflammatory cytokines, e.g., IL-4, IL-11, IL-13, and IL-37, and many of these become enriched in certain tissues (Human Protein Atlas). However, the expression of their receptors has low tissue specificity, e.g., IL-4R and IL-11R. IL-4 and IL-13 cytokines control the JAK/STAT6 signaling through the co-operation of IL-4R and IL-13R. Both immune and several non-immune cells can secrete IL-4/IL-13 cytokines and furthermore they can respond to these cytokines [139]. The IL-4/IL-13 axis can induce a broad range of responses in different cells, e.g., stimulating Th2 differentiation and the M2 polarization of macrophages. Many inflammatory diseases are associated with dysfunctions in IL-4/IL-13 signaling [139]. Interestingly, IL-4 exposure can induce many alterations linked to the aging process, e.g., cardiac fibrosis [140] and cellular senescence [141]. IL-11 is an anti-inflammatory member of IL-6 cytokine family, which can activate both the JAK/STAT3 signaling and the PI3K/AKT/mTOR axis [142]. Given that IL-11 activates STAT3 signaling, Sumida et al. [143] demonstrated that IL-11 induced the differentiation of monocytic MDSCs involving the upregulation of ARG1 and enhanced capacity to suppress T cell proliferation. Currently, the functions of tissue-enriched anti-inflammatory cytokines are poorly understood.

#### Reactive oxygen and nitrogen species

Immunosuppressive cells secrete reactive oxygen (ROS) and nitrogen (RNS) species with which they communicate within the immunosuppressive network and they also exploit these compounds in the suppression of effector immune cells [61, 144, 145]. NADPH oxidases (NOX), nitric oxide synthases (NOS and iNOS), and mitochondrial electron transfer chain are common sources of ROS/RNS generation in immune cells. For instance, Xu et al. [146] demonstrated that the ROS production by NOX1 and NOX2 induced the differentiation of mouse monocytes into macrophages and consequently enhanced their polarization into the M2 and tumor-associated macrophages (TAM), two immunosuppressive phenotypes of macrophages. Roux et al. [147] revealed that increased ROS level activated NF-κB signaling which promoted the expression of PD-L1 protein and the release of immunosuppressive chemokines in mouse and human macrophages. Moreover, it is known that the NOX-stimulated ROS production from macrophages induced the generation of Tregs in rats and humans [145]. The activation of MDSCs in different tumor models stimulated the expression of NOX2 which enhanced ROS production and T cell suppression [144]. Nagaraj et al. [148] reported that ROS and NO produced by mouse MDSCs nitrated the tyrosine residues of the T cell receptor (TCR) in CD8^+^ T cells. This nitration of the TCR prevented the antigen-specific stimulation of these cells. MDSCs can also impair the activity of NK cells and the antigen presentation of dendritic cells through the production of NO [149, 150]. Given that immunosuppressive cells generate ROS/RNS compounds, it is essential that they are protected against oxidative stress. Beury et al. [151] demonstrated that mouse MDSCs were resistant to ROS although they released an elevated level of ROS compounds. They reported that the increased expression of NRF2, a transcription factor for the antioxidant enzymes, improved the survival of MDSCs in inflamed mouse tumor conditions. The expression of NRF2 reduced the rate of apoptosis of MDSCs which increased the numbers of MDSCs in tumors. Aging and age-related diseases are associated with increased oxidative stress in tissues which might augment the activity of the immunosuppressive network and consequently enhance tissue degeneration with aging.

ROS compounds are able to control several signaling pathways by affecting protein kinases and other cell-signaling proteins [152]. One especially interesting target is the co-operation between ROS and the TGF-β-mediated processes, e.g., fibrosis, cellular senescence, and apoptosis [153, 154]. For instance, there is robust evidence that TGF-β signaling promoted the mitochondrial and the NOX-mediated ROS generation [154]. TGF-β stimulates mitochondrial ROS production by suppressing the expression of adenine nucleotide translocase-2 (ANT2) [155] and also reducing the activity of complex IV in the electron transfer chain [156]. Increased mitochondrial ROS generation is known to provoke cellular senescence. It seems that there exists a feed-forward mechanism since ROS stimulate the expression and activity of TGF-β and consequently enhance the responses mediated by TGF-β signaling in host tissues. For instance, the TGF-β-induced muscle atrophy is a ROS-dependent process [157] as well as that of lung fibrosis [158]. There are very few observations on the role of ROS in the control of IL-10-mediated responses, e.g., ROS enhanced the responses induced by IL-10 signaling in endotoxin-induced lung inflammation in mice [159]. Interestingly, the age-related oxidative stress in host tissues might be a potent enhancer for the responses induced by TGF-β and IL-10 signaling and thus aggravate tissue degeneration.

There is compelling evidence that the age-related low-grade inflammation in tissues is associated with increased oxidative stress. However, currently, it is not known what is the source of ROS generation in host tissues in the inflammaging process. Moreover, as discussed above, oxidative stress can enhance the activity of immunosuppressive cells and concurrently increase the production of ROS and RNS compounds which consequently induce detrimental bystander effects in host tissues during chronic inflammation. However, ROS and RNS can have both beneficial and harmful effects in a context-dependent manner on human health and lifespan [160, 161]. ROS and RNS compounds are very reactive molecules which can damage host tissues through the oxidative modification of a diverse set of molecules, e.g., carbonylation, S-nitrosylation, S-glutathionylation, and sulfoxidation. It seems that there is a balance between specific, physiological redox signaling and excessive, pathological reactions [162]. However, the aging process and many age-related diseases elicit an increase in the level of oxidized molecules which can provoke the accumulation of protein/lipid aggregates, e.g., lipofuscin. Notably, superoxide and NO can react with each other and generate very active peroxynitrite (OONO^−^) compound. For instance, peroxynitrite can nitrate the T cell receptor and disturb the function of T cells [148]. Moreover, van der Loo et al. [163] reported that increased ROS generation with aging reduced NO content in rat endothelial cells through the formation of peroxynitrites, thus enhancing a process which promoted vascular aging.

On the other hand, immunosuppressive cells can downregulate the generation of NO which has crucial functions in cardiovascular health and diseases [36, 164]. For instance, NO controls vasodilatation through the cooperation of vascular endothelium and smooth muscles. NO also inhibits the adhesion of platelets, leukocytes, and monocytes to endothelium as well as it prevents the hyperplasia of blood vessel intima. Accordingly, disturbances in NO signaling can provoke cardiovascular disorders, e.g., hypertension and atherosclerosis. l-arginine is a shared substrate for both the NOS enzymes and ARG1 [36] which means that there exists a competition for l-arginine in inflamed tissues. Given that the increased expression of ARG1 and the subsequent depletion of l-arginine are a potent mechanism used by immunosuppressive cells to inhibit inflammation (see below), it is likely that the activation of immunosuppressive network, such as what occurs during aging [8], causes a deficiency of NO synthesis and thus elicits vascular disorders (Figs. 2 and 3). There are studies indicating that the increase in the activity of ARG1 with aging promotes vascular stiffness [165]. White et al. [166] reported that the knockdown of ARG1 restored NO signaling and improved vasodilatory effects in the aorta of old rats. Zhu et al. [167] demonstrated that the overexpression of ARG1 in human endothelial cells induced the uncoupling of eNOS and consequently increased the expression of inflammatory and senescence markers. NO deficiency might also promote the pathology of Alzheimer’s disease. Austin et al. [168] reported that transgenic eNOS^−/−^ mice displayed an increased level of inflammatory markers as well as augmented the expression and processing of amyloid precursor protein (APP) in mouse hippocampus. It seems that the activation of immunosuppressive cells can disturb the balance between the activities of ARG1 and NOS enzymes and subsequently this can lead to vascular disorders.

### Amino acid catabolism

Immunosuppressive cells exploit the auxotrophy of many immune cells for distinct amino acids, e.g., l-arginine and tryptophan, in the generation of immunosuppression. Auxotrophy means that cells are unable to synthesize a particular amino acid which is essential for their function. Regulatory immune cells deplete the microenvironment from these amino acids to suppress innate and adaptive immune responses [169]. However, the shortage of amino acids also induces bystander effects in neighboring cells although many cell types can activate the synthesis of the depleted amino acid. Several immunosuppressive cells express ARG1 which exhausts the l-arginine stored in the microenvironment [170]. Another immunosuppressive mechanism is the expression of indoleamine 2,3-dioxygenase (IDO) which depletes tryptophan from inflamed environments [171]. It is not only the starvation of amino acids from the local microenvironment but also concurrently these regulatory immune cells generate immunomodulatory metabolites, e.g., IDO activates the kynurenine pathway [172] (Fig. 3).

### ARG1 and arginine metabolism

l-arginine is a conditionally essential amino acid which is involved in pivotal functions in cellular metabolism. The ARG1-induced depletion of l-arginine from the microenvironment is a common mechanism of myeloid suppressor cells to prevent the proliferation and function of T cells which display an l-arginine auxotrophy [169, 173]. In some myeloid suppressor cells, the activation of NOS enzymes also triggers the exhaustion of l-arginine from the milieu. For instance, M-MDSCs display an increased ARG1 expression, whereas PMN-MDSCs show an elevated level of iNOS [174]. M2 macrophages display an increased expression of ARG1 but show a negligible expression of iNOS. l-arginine depletion mainly affects the cells which express l-arginine auxotrophy. However, the expression of argininosuccinate synthetase (ASS), catalyzing the de novo synthesis of l-arginine, seems to be very low in many tissues (Human Protein Atlas). This means that the deprivation of l-arginine might not only affect l-arginine-deficient immune cells but also disturb the functions of neighboring cells. Interestingly, there are some tumor types which are dependent on extracellular l-arginine since they do not express ASS [175]. ARG1 and NOS enzymes are not only catabolic enzymes which can process/compete from the tissue pool of l-arginine. Arginine decarboxylase generates agmatine which subsequently can be metabolized to urea and polyamines. Agmatine has many neuroprotective effects [176]. The activation of ARG1 and iNOS most probably impairs the generation of agmatine in inflamed tissues. The most important l-arginine catabolic pathways are the arginine/NO pathway and the arginine/ornithine pathway [36]. The effects of NO on the health of host tissues were discussed above. The activation of ARG1 produces ornithine and consequently common polyamines, i.e., putrescine, spermidine, and spermine. Polyamines exert many beneficial effects on both immune cells and host tissues. For instance, spermidine increases autophagy and thus it has important cardioprotective and neuroprotective effects [177]. However, the increased level of polyamines has many pathological effects, e.g., on cancer progression and neurological diseases [178]. For instance, the catabolism of polyamines generates ROS compounds which might have an important role in the generation of immunosuppression but oxidative stress commonly exerts detrimental effects on the host tissues. Currently, it is not known whether polyamines induce immunosuppression although there are studies indicating that polyamine-blocking therapy might reverse immunosuppressive conditions, e.g., by reducing the level of MDSCs and increasing the numbers of CD3^+^ T cells, in a tumor microenvironment in mice [179].

The maintenance of tissue amino acid homeostasis has a crucial role for the growth, metabolism, and survival of organism. There are two major sensor mechanisms which assess the balance of amino acids in tissues, i.e., the mechanistic target of rapamycin complex 1 (mTORC1) and general control nonderepressible 2 (GCN2) (Fig. 3). The GCN2 kinase is activated by amino acid deprivation and it is linked to the regulation of eIF2α-driven protein synthesis and gene expression (see below). mTORC1 is the nutrient-sensing protein kinase which regulates both the growth and metabolism in eukaryotes [180]. The mTORC1 complex acts as a sensor for the cellular availability of arginine, leucine, and methionine through different mechanisms. Chantranupong et al. [181] demonstrated that the CASTOR1 protein detected the presence of arginine in the cytoplasm of mammalian cells. The binding of arginine to the CASTOR1 protein stimulated downstream mTORC1 kinase through the GATOR-RagGTPase complex at the lysosomal surface. The deprivation of arginine prevented the activation of mTORC1 kinase. Moreover, the SLC38A9 protein, an arginine sensor in the lysosomal compartment, activates mTORC1 kinase and also acts as the arginine-dependent transporter of essential amino acids from lysosomes [182]. This means that arginine is the fundamental regulator of mTORC1 activity and thus it controls protein synthesis and diverse metabolic responses in mammalian cells. Interestingly, there is substantial evidence that the nutrient-sensing mTORC1 pathway has a key role in the regulation of aging and longevity [183]. It is known that dietary restriction linked to reduced amino acid intake extends health span and longevity; this phenomenon is evident from yeast to humans [184]. In fact, the increased activity of mTORC1 reduces lifespan, whereas the reduced activity of mTORC1, i.e., amino acid shortage, increases health span and longevity. There is abundant evidence indicating that protein synthesis is reduced with aging [185]. Correspondingly, protein turnover time is extended, especially due to a slowing of autophagy, which means that cellular protein quality control deteriorates with aging. Currently, it is very likely that the deprivation of arginine exerts bystander effects on the activity of mTORC1 kinase in inflamed microenvironments.

### IDO and kynurenine signaling

IDO is the rate-limiting enzyme in the kynurenine pathway which catabolizes tryptophan, an essential amino acid, to kynurenine and a number of intermediate metabolites. Ultimately, these metabolites can be processed to nicotinamide adenine dinucleotide (NAD) [37, 172]. The inflammatory mediators are the potent inducers of IDO expression, especially IFN-γ and TGF-β. IDO has not only been expressed in immunoregulatory cells but many non-immune cells display the expression of IDO, e.g., many cancer cells. The activation of IDO in inflamed tissue induces a robust immunosuppressive response [34, 186]. For instance, Holmgaard et al. [186] demonstrated that the expression of IDO in human and mouse tumors induced the recruitment, expansion, and activation of MDSCs in tumor sites. They observed that the activation of MDSCs was dependent on the presence of FoxP3^+^-positive Tregs. Recently, Ladomersky et al. [187] reported that the age-related decrease in the efficacy of immunotherapy against mouse glioblastoma was associated with an increase in the expression of IDO protein with aging in mouse brain. However, the age-related immunosuppression was not reversed by the treatment with the pharmacological inhibitor of IDO enzyme. Interestingly, Mezrich et al. [188] demonstrated that kynurenine, the first metabolite of tryptophan degradation, activated aryl hydrocarbon receptor (AhR) signaling. The activation of AhR, a transcription factor, induced the differentiation of mouse CD4^+^ T cells into the immunosuppressive FoxP3^+^ Tregs. They also revealed that TGF-β stimulated the expression of AhR and accordingly, the activation of AhR stimulated the IDO expression in mouse dendritic cells. MDSCs and Tregs display an elevated expression of IDO which enhances immunosuppression in inflamed tissues by activating the function of the immunosuppressive network.

The activation of IDO induces the deprivation of tryptophan in inflamed tissues (Fig. 3). The deficiency of this amino acid stimulates GCN2 kinase through the accumulation of uncharged tRNAs into the cell [189]. GCN2 phosphorylates the eukaryotic initiation factor 2α (eIF2α) which consequently inhibits protein synthesis. Given that tryptophan is an essential amino acid, it is expected that the activation of IDO as well as the tryptophan transporters in immunosuppressive cells can induce a tryptophan depletion in inflamed tissue which leads to an inhibition of protein synthesis in nearby non-immune cells. Clear tissue atrophy is evident not only in aged tissues but also in many age-related diseases, e.g., Alzheimer’s disease [190]. The activation of GCN2 also controls gene expression since phospho-eIF2α stimulates the activating transcription factor 4 (ATF4) [191]. The activation of ATF4 can cause many pathological effects in tissues although it is able to increase cellular survival during stressful situations. For instance, the activation of ATF4 can promote skeletal muscle atrophy [192], atherosclerotic calcification of vascular smooth muscles [193], and neurodegenerative diseases [194]. There are several studies indicating that the increased activity of eIF2α/ATF4 signaling exerts detrimental effects in Alzheimer’s pathology, e.g., it increases the generation of β-amyloid peptides and tau-protein phosphorylation [195]. On the other hand, there is an abundant literature that the deficiency of essential amino acids, especially that of tryptophan and methionine, can increase the lifespan of several species, e.g., mammals [196]. Currently, the detailed mechanism is still unknown although it is known that the activation of ATF4 via GCN2 stimulates integrated stress response (ISR) which increases the stress resistance of the organism [197] (Fig. 3).

The inflammation-induced activation of IDO in immune cells generates kynurenine and its metabolites which affect the functions of neighboring cells. In addition to beneficial effects, e.g., blood vessel dilatation, kynurenines are involved in a number of pathological processes, e.g., cardiovascular diseases [198], neurodegenerative diseases [199], muscle atrophy [200], and osteoporosis [201]. There are observations that kynurenines might have a significant role in the inflammaging process, e.g., in the control of inflammatory responses [202]. Moreover, Guillemin et al. [203] reported that the expression of IDO and the production of the excitotoxic compound, quinolinic acid, which is a metabolite of the kynurenine pathway, were robustly increased in the hippocampus of AD patients. The pathological mechanisms induced by kynurenines are poorly understood. It is known that the kynurenine pathway and its metabolites affect the redox pathways and energy metabolism [204, 205]. Wang et al. [206] demonstrated that tryptophan-derived 3-hydroxykynurenine stimulated the activity of NADPH oxidase (NOX) and increased superoxide production inducing dysfunctions and apoptosis in mouse endothelial cells. Kynurenine also inhibited autophagy and promoted senescence, e.g., in the mesenchymal stem cells isolated from the bone marrow of old mice [207]. Given that serotonin is synthesized from tryptophan, the depletion of tryptophan in chronic inflammation can provoke metabolic, psychiatric, and gastrointestinal disorders [208]. It seems that the function of IDO not only induces immunosuppression via the tryptophan catabolism but the breakdown of tryptophan through the kynurenine pathway triggers many of the pathological processes commonly observed during aging although causality needs to be clarified.

## Conclusions

### Is immunosuppression the driving force in the aging process?

There is convincing evidence that chronic low-grade inflammation is associated with the aging process in many different species. Concurrently with inflammatory changes, the signs of senescence, i.e., a state called immunosenescence, appear in the immune system. Currently, it is not known whether the remodeling of the immune system associated with aging is the cause or consequence of the chronic inflammation. The origin of age-related inflammation still needs to be clarified although there are several possible sources. Chronic inflammation is commonly associated with the compensatory immunosuppressive response. There is substantial evidence that immunosuppression increases with aging [8]. Interestingly, the hallmarks of immunosenescence are similar to those induced by immunosuppressive MDSCs, e.g., (i) a decline in the functions of T and B cells, (ii) a decrease in antigen presentation by dendritic cells, and (iii) the inhibition of cytotoxicity of NK cells [209]. Given the close cooperation between the immune cells within the immunosuppressive network, it seems likely that age-related chronic inflammation activates the immunosuppressive network which remodels the immune system with aging promoting immunosenescence with many harmful effects, e.g., increased risk for cancers, enhanced vulnerability to infections, and decreased vaccination efficacy. However, the activation of immunosuppressive network keeps pro-inflammatory cells under control, thus preventing tissue damage induced by persistent inflammation.

The armament of immunosuppressive cells utilizes both the targeted and non-targeted mechanisms to suppress the activity of effector immune cells. Immunosuppressive cells, e.g., MDSCs and Tregs, exploit the contact-dependent immune checkpoint receptors, such as PD-L1/PD-1 and CTLA-4 systems, to inhibit the function of immune cells [210]. However, the majority of the weapons are soluble tools, e.g., TGF-β and IL-10 cytokines and ROS, as well as alterations in the catabolism of arginine and tryptophan in inflammatory conditions. These non-targeted mechanisms also affect the neighboring cells in distressed tissues and provoke degenerative changes if the inflammation becomes chronic. For instance, the receptors of TGF-β and IL-10 are widely expressed in non-immune cells and especially TGF-β signaling can trigger many age-related degenerative changes, e.g., cellular senescence, fibrosis, osteoporosis, and ECM disruption. Moreover, the secretion of toxic ROS/RNS compounds damage host tissues in chronic inflammation. Immunosuppressive cells also exploit amino acid catabolism which has direct effects on the nearby cells via the depletion of amino acids, e.g., the shortage of tryptophan, an essential amino acid, might enhance atrophic degeneration in inflamed tissues. The ARG1-induced deprivation of NO might enhance vascular aging, whereas the IDO-induced kynurenine metabolites are able extensively to disturb tissue homeostasis. In conclusion, the age-related low-grade inflammation activates compensatory immunosuppression which not only triggers immunosenescence but also disturbs tissue homeostasis, thus enhancing tissue degeneration with aging.

## Data Availability

Not applicable
